# Novel *RAB27A* Variant Associated with Late-Onset Hemophagocytic Lymphohistiocytosis Alters Effector Protein Binding

**DOI:** 10.1007/s10875-022-01315-4

**Published:** 2022-07-23

**Authors:** Timo C. E. Zondag, Lamberto Torralba-Raga, Jan A. M. Van Laar, Maud A. W. Hermans, Arjen Bouman, Iris H. I. M. Hollink, P. Martin Van Hagen, Deborah A. Briggs, Alistair N. Hume, Yenan T. Bryceson

**Affiliations:** 1 Department of Internal Medicine, Section Allergy & Clinical Immunology, Erasmus University Medical Center Rotterdam, Rotterdam, the Netherlands; 2 Department of Immunology, Erasmus University Medical Center Rotterdam, Rotterdam, the Netherlands; 3 Center for Hematology and Regenerative Medicine, Department of Medicine, Huddinge, Karolinska Institute, Stockholm, Sweden; 4 Department of Clinical Genetics, Erasmus University Medical Center Rotterdam, Rotterdam, the Netherlands; 5 Faculty of Medicine & Health Sciences, University of Nottingham, Nottingham, UK; 6 Department of Clinical Immunology and Transfusion Medicine, Karolinska University Hospital, Stockholm, Sweden; 7 Broegelmann Research Laboratory, Department of Clinical Science, University of Bergen, Bergen, Norway

**Keywords:** Griscelli syndrome type 2, Hemophagocytic lymphohistiocytosis, Lymphocyte cytotoxicity, Inborn errors of immunity, Late-onset

## Abstract

Autosomal recessive mutations in *RAB27A* are associated with Griscelli syndrome type 2 (GS2), characterized by hypopigmentation and development of early-onset, potentially fatal hemophagocytic lymphohistiocytosis (HLH). We describe a 35-year old male who presented with recurrent fever, was diagnosed with Epstein-Barr virus-driven chronic lymphoproliferation, fulfilled clinical HLH criteria, and who carried a novel homozygous *RAB27A* c.551G > A p.(R184Q) variant. We aimed to evaluate the contribution of the identified *RAB27A* variant in regard to the clinical phenotype as well as cellular and biochemical function. The patient displayed normal pigmentation as well as RAB27A expression in blood-derived cells. However, patient NK and CD8^+^ T cell exocytosis was low. Ectopic expression of the RAB27A p.R184Q variant rescued melanosome distribution in mouse Rab27a-deficient melanocytes, but failed to increase exocytosis upon reconstitution of human RAB27A-deficient CD8^+^ T cells. Mechanistically, the RAB27A p.R184Q variant displayed reduced binding to SLP2A but augmented binding to MUNC13-4, two key effector proteins in immune cells. MUNC13-4 binding was particularly strong to an inactive RAB27A p.T23N/p.R184Q double mutant. RAB27A p.R184Q was expressed and could facilitate melanosome trafficking, but did not support lymphocyte exocytosis. The HLH-associated RAB27A variant increased Munc13-4 binding, potentially representing a novel mode of impairing RAB27A function selectively in hematopoietic cells.

**Supplementary Information:**

The online version contains supplementary material available at 10.1007/s10875-022-01315-4.

## Introduction

Griscelli syndrome type 2 (GS2) is a pigmentation disorder associated with autosomal recessive mutations in *RAB27A* [26]. In contrast to other forms of GS, GS2 patients typically develop early-onset, life-threatening hemophagocytic lymphohistiocytosis (HLH), a hyperinflammatory syndrome [40]. Familial forms of HLH are associated with defective lymphocyte cytotoxicity, which requires exocytosis of cytotoxic granules, a form of specialized lysosomes [8].

*RAB27A* encodes RAB27A, a 25 kDa member of the Rab family of small GTPases [28]. The C-terminus can be prenylated by Rab geranylgeranyltransferase (RGGTase) acting on cysteine-containing motifs, thereby anchoring RAB27A to the membrane [21, 33, 34]. GTPase are activated by guanin exchange factors (GEFs), which induce an active, effector protein-binding conformation through exchange of GDP for GTP. In turn, GTPase-activating proteins (GAPs) inactivate GTPases [2]. These forms are mimicked by RAB27A Q78L (active) and T23N (inactive) substitutions. In melanocytes, the dispersal of pigment-containing melanosomes is driven by RAB27A, which coordinates the melanophilin-myosin-Va motor complex and an actin filament assembly complex, as a prelude to melanin exocytosis [1, 40]. In hematopoietic cells, secretory lysosome trafficking, docking, and exocytosis is mediated by RAB27A interactions with SLP2A and MUNC13-4 [9, 12]. The RAB27A/SLP2A complex has been crystalized, revealing that the RAB27A α5-helix interacts with SLP2A [5]. In contrast, HLH-associated RAB27A missense variants that disrupt MUNC13-4 binding have been mapped to the RAB27A α4-helix [4]. The RAB27A interaction with MELANOPHILIN has not been mapped but does not interfere with MUNC13-4 binding [4]. These observations can explain how certain previously reported RAB27A variants specifically impair MUNC13-4 binding and exocytosis in hematopoietic cells, without affecting pigmentation in melanocytes [4, 31].

We describe an adult-onset HLH patient from consanguineous parents harboring a novel homozygous *RAB27A* c.551G > A p.(R184Q) variant. Our results suggest a novel mode of selective disruption of RAB27A function in hematopoietic cells.

## Methods

### Patient and Control Samples

This study was approved by the ethic committees of the Board of Stockholm. Informed consents from the individuals included in the study were obtained according to the Declaration of Helsinki. The patient was diagnosed according to the HLH-2004 criteria. Clinical data, laboratory findings, and genetics were collected from the patient’s medical records. Peripheral blood mononuclear cells (PBMCs) and hair were collected and analyzed. Six siblings were unavailable or did not consent to genetic analyses.

### DNA Extraction, Amplification, and Sequence Analysis

DNA was enriched using Agilent SureSelect Clinical Research Exome V2 capture and paired‐end sequenced on the Illumina platform. The aim was to obtain 8.1 Giga base pairs per exome with a mapped fraction of 0.99. The average coverage of the exome was ~ 50 × . Duplicate reads were excluded. Data were demultiplexed with bcl2fastq Conversion Software from Illumina. Reads were mapped to the genome using the BWA‐MEM algorithm (reference: http://bio‐bwa.sourceforge.net/). Variant detection was performed by the Genome Analysis Toolkit HaplotypeCaller (reference: http://www.broadinstitute.org/gatk/). The detected variants were filtered and annotated with Cartagenia software and classified with Alamut Visual.

Sequence variants were searched in a primary immunodeficiency panel covering > 300 genes. Homozygous *VPS13B* c.2471C > T p.(S824F) and heterozygous *CARD11* c.2711G > A p.(S904N) variants of uncertain significance were also identified. No known pathogenic variants were identified.

### Immunophenotyping and Cytotoxic Lymphocyte Function Analysis

Lymphocyte subset numbers were quantified by flow cytometry (FACS Symphony instrument, BD Biosciences) using BD IMK kit with TruCount tubes (BD Biosciences) according to the manufacturer’s instructions. Lymphocyte phenotype and function were further assessed upon stimulation and staining of freshly isolated PBMC [7]. Briefly, fluorochrome-conjugated anti-CD3 (BioLegend), anti-CD4 (Invitrogen), anti-CD8 (BioLegend), anti-CD16 (BD Bioscience), anti-CD56 (BD Bioscience), and anti-CD107a (BioLegend) monoclonal antibodies were used. Functional testing of cytotoxic lymphocytes was performed incubating PBMC in vitro with murine P815 cells together with anti-CD16 or anti-CD3 antibodies for stimulation of NK cells and T cells, respectively. Natural cytotoxicity was tested using K562 cells. Exocytosis was quantified using CD107a^+^ surface expression. Flowjo v.9.9 (BD Biosciences) was used for analysis of the flow data.

### Western Blot for RAB27A in Primary Cells

One million PBMCs per donor were lysed in RIPA buffer supplemented with 1 × protease inhibitor cocktail (Santa Cruz Biotechnology) for 30 min on ice. Supernatants were mixed with 4 × NuPage loading buffer (Invitrogen) added 10 mM DTT (Invitrogen), run on a 4–12% Bis–Tris gel (Invitrogen), and transferred to a nitrocellulose membrane (iBlot, Invitrogen). Rabbit polyclonal anti-RAB27A (Proteintech Group) and HRP-conjugated goat anti-rabbit secondary antibodies (Invitrogen) were used for detection. A directly HRP-conjugated mouse anti-actin antibody (Sigma) was used as loading control. Blocking buffer and antibodies were diluted in 5% non-fat dry milk (Biorad) in TBS-Tween 0.2%.

### Sequence Alignment and 3D Structure Visualization

RAB27A protein sequences of different organisms were downloaded from the NCBI database (https://www.ncbi.nlm.nih.gov/) and aligned using CLC Main Workbench software (version 7.0, Qiagen). The 3D structure of RAB27A interacting with SLP2A (PDB 3BC1) was downloaded and visualized using Chimera 1.13 software.

### Plasmid Constructs

Plasmids encoding *RAB27A* WT, p.Q78L, and p.T23N were kindly provided by Dr. Genevieve de Saint Basile [24]. PCR amplification was performed to shuttle cDNA to a modified pMax backbone with an N-terminal 3xFLAG tag using *Nhe*I and *Age*I restriction sites. The *RAB27A* c. 551G > A (p.R184Q) variants were generated by site-directed mutagenesis. The plasmid sequences were confirmed by Sanger sequencing. A SLP2A-hem containing vector was kindly provided by Dr. G. de Saint Basile and transferred to a vector with an N-terminal MYC-tag [25]. N-terminal MYC-tagged MUNC13-4 and MELANOPHILIN constructs were also generated. Adenovirus vectors allowing expression of the RAB27A p.R184Q mutant as a fusion to monomeric red fluorescent protein (mRFP) were generated as previously described [14]. For the lentiviral constructs, RAB27A WT and p.R184Q were cloned into pLeGO-G2 (Addgene plasmid #25,917) using *Bam*HI and *Eco*RI restriction sites. Later, viral particles (VSV-G) from supernatant of packing HEK-293FT cells were added to stimulated CD8^+^ T cells from healthy individuals or GS2 patients.

### Melanosome Distribution

Immortal Rab27a-deficient murine ashen melanocytes were cultured as previously described [13]. For analysis of melanosome distribution, 2.5 × 10^4^ cells were plated on 13-mm glass coverslips. Twenty-four hours later, the cells were transduced with adenovirus expression vectors, and after a further 24 h of incubation, these were fixed and stained to detect the localization of RAB27A wild-type and p.R184Q proteins as previously described [13]. Intracellular distribution of melanosomes and RAB27A was recorded as previously described [13].

### Reconstitution Experiments in CD8.^+^ T Cells from RAB27A-Deficient Patients

CD8^+^ T cells were isolated from PBMC of selected GS2 patients by negative magnetic selection (Miltenyi Biotech), stimulated with 10 µL anti-CD3/CD28 immunocomplexes (STEMCELL Technologies) and 100 IU/mL of recombinant IL-2 for 48 h. Cells were thereafter transduced with VSV-G viral particles containing constructs encoding either N-terminally mCherry tagged RAB27A wild-type or p.R184Q. The next day, the cells were washed and cultured in complete medium supplemented with 100 UI/mL IL-2. After 3 days of culture, cells were assessed for exocytosis by surface expression of CD107a as previously described. Cells were cultured in RPMI medium (Hyclone) supplemented with 10% FCS at 37 °C, 5% CO_2_.

### Co-immunoprecipitation of Effector Proteins

HEK-293FT cells were chemically transfected (Lipofectamine 2000, Invitrogen) according to the manufacturers’ protocol. After 24 h, cells were lysed in 25 mM Tris–HCl pH 8.0, 150 mM NaCl, 1 × protease inhibitor cocktail (HALT), 250 U/mL benzonase (Invitrogen), 10 mM DTT, 1% TritonX-100, 5 mM EDTA, and 0.5 × sodium orthovanadate. A goat anti-FLAG tag antibody (Abcam) was used for immunoprecipitation, with magnetic protein G beads used to harvest the immunocomplexes (Dynabeads, Thermo). This was then eluted in the presence of NuPAGE LDS buffer (Invitrogen), run on a 4–12% Bis–Tris gel (Invitrogen), and transferred to a nitrocellulose membrane (iBlot, invitrogen). Mouse anti-Myc (Invitrogen) and mouse anti-FLAG (Sigma) antibodies were used to blot for the recombinant proteins.

### Statistical Analysis

Mean values, standard deviation, and *p*-values (paired parametric *t*-test) were calculated using GraphPad Prism 7.0 software (GraphPad Prism Inc.). The threshold for statistical significance was set at *p* ≤ 0.05.

## Results

### A Novel Homozygous RAB27A p.(R184Q) Variant Identified in a HLH Patient

**Fig. 1:**
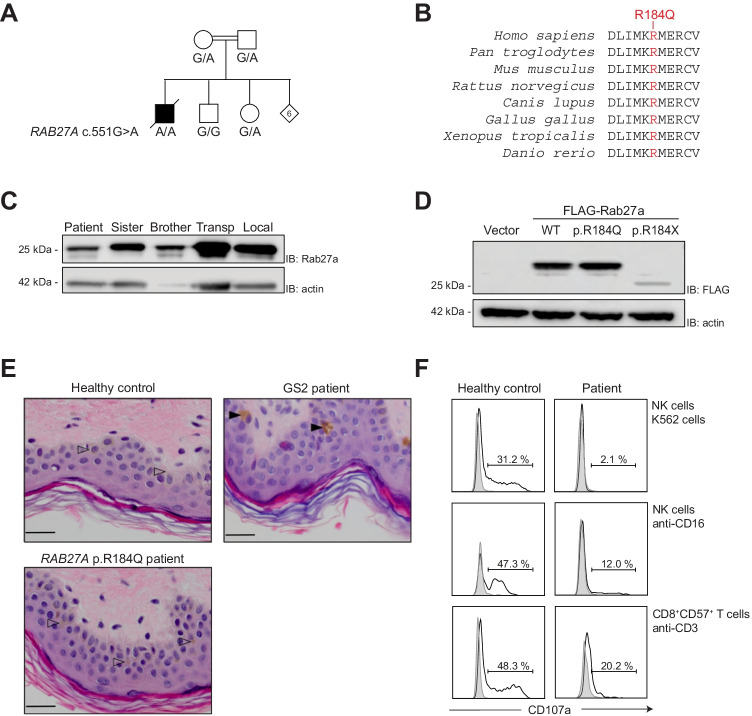
A novel homozygous *RAB27A* c.551G > A (p.R184Q) variant in a fatal HLH case. **A** Pedigree of family. Six out of eight siblings were not available for genetic analysis. **B** RAB27A amino acid evolutionary conservation in mammals, birds, frogs, and fish of the sequence surrounding the p.R184Q variant. **C** Expression of RAB27A determined by western blot of freshly isolated PBMC lysates from the patient, siblings, and healthy controls, as indicated. Actin was used as loading control. Blots are representative of two independent experiments. **D** Western blot of HEK-293FT cells transiently transfected with plasmids encoding FLAG-RAB27A wild-type (WT), patient-derived p.R184Q, or truncating p.R184X variants. Results are representative of three independent experiments. **E** Hematoxylin–eosin staining of skin biopsies from a healthy control (indicating normal melanocytes with arrowheads), a typical GS patient (displaying characteristic hyperpigmented oval melanocytes indicated with filled arrowheads), and the patient. Bars indicate 20 mm. **F** Histograms show exocytosis (quantified on the basis of CD107a surface expression) of cytotoxic lymphocyte subsets from the patient as well as a healthy transport control, as specified. PBMCs were stimulated with target cells and antibodies as indicated, for 2 h. The cells were analyzed by flow cytometry, gating on CD3^–^CD56^+^ NK cells or CD3^+^CD8^+^CD57.^+^ T cells. Data are representative of two independent experiments

A 35-year-old male with a history of recurrent sinopulmonary infections and schizophrenia initially presented with recurrent fever and dry coughs. He was from consanguineous parents of Turkish origin and had eight siblings (Fig. 1A). He was initially diagnosed with EBV-driven lymphoproliferation based on high EBV copy numbers (9929 IU/mL) and pathology. In spite of rituximab therapy, the fever persisted. Initially, only a mild anemia was present and ferritin levels were low. However, 3 months after the diagnosis of chronically active EBV disease, the patient developed overt inflammation, fulfilling the HLH-2004 criteria (Table 1) [11]. Ferritin peaked at 67,938 μg/L. Despite extensive efforts, lymphoma was excluded, and no other underlying cause of HLH besides EBV infection was identified. The patient was treated with corticosteroids, intravenous immunoglobulin, etoposide, rituximab, and alemtuzumab, but the HLH repeatedly relapsed. Almost 2 years after initial presentation with EBV-driven lymphoproliferation, the patient developed pulmonary aspergillosis and died of pulmonary insufficiency in anticipation of a hematopoietic stem cell transplant.

**Table 1:** HLH-2004 criteria at diagnosis

Fever	Yes
Splenomegaly	Yes
Cytopenias (affecting ≥ 2 of 3 lineages)	
Haemoglobin < 90 g/L	77
Platelets < 100 × 10^9^/L	75
Neutrophils < 1.0 × 10^9^/L	2.17
Hypertriglyceridemia and/or hypofibrinogenemia	
Fasting triglycerides ≥ 3.0 mmol/L	3.07
Fibrinogen ≤ 1.5 g/L	5.4
Hemophagocytosis in bone marrow or spleen or lymph nodes	Yes
Low or absent NK-cell activity	Yes
Ferritin ≥ 500 µg/L	2135
sIL-2 receptor ≥ 2400U/ml	82,606

Whole-exome sequencing uncovered a homozygous missense variant in *RAB27A*:NM_004580.4 (RAB27A):c.551G > A, p.(R184Q) (Fig. 1A), which has a population frequency of < 0.0001 according to public databases (gnomAD v3.1.1) [15], is predicted damaging (CADD score 25.20) [16, 35], and has not previously been associated with HLH. Representing a change from a positively to a negatively charged amino acid in the C-terminal α5-helix of RAB27A, the R184 position is highly conserved among vertebrates (Fig. 1B). In addition, rare homozygous *VPS13B* c.2471C > T p.(S824F) and a heterozygous *CARD11* c.2711G > A p.(S904N) variants of uncertain significance were also identified (CADD scores 3.54 and 23.0, respectively). Autosomal recessive *VPS13B* variants cause Cohen syndrome, characterized by obesity, hypotonia, mental deficiency, and facial, oral, ocular, and limb anomalies [19]. Leukopenia, especially neutropenia, is also a feature of Cohen syndrome [30]. Apart from mild cognitive impairment, the patient did not present clinical features characteristic of Cohen syndrome illustrated by a body mass index (BMI) between 20 and 25, normal muscle tone, absent psychomotor retardation, and no syndromic appearances/anomalies. Germline *CARD11* mutations are associated with different primary immune disorders in humans [22]. The patient’s history of recurrent sinopulmonary infections and persistent EBV infection overlaps with clinical manifestations of heterozygous CARD11 mutations causing B-cell expansion with NF-κB and T-cell anergy (BENTA). Of note, heterozygous *CARD11* variants associated with BENTA are typically located in the N-terminal CARD and LATCH domains and not in the C-terminus as was the case in this patient. In addition, B-cell expansions were not observed in our patient. Given a paucity of features associated with Cohen syndrome, yet association of autosomal recessive *RAB27A* variants with HLH, we focused further on evaluating the potential contribution of the predicted damaging *RAB27A* variant to disease.

### Rab27a Expression and Patient Characteristics

In order to examine the expression of the RAB27A variant protein, we performed western blots of peripheral blood mononuclear cell lysates. The patient expressed RAB27A (Fig. 1C), indicating that the protein was not degraded. Furthermore, ectopic expression of RAB27A wild-type (WT), p.R184Q, and p.R184X constructs in 293FT cells also revealed comparable expression of RAB27A WT and p.R184Q, whereas the p.R184X was degraded (Fig. 1D). The RAB27A p.R184X mutant cannot be C-terminally prenylated and hence is unstable [27].

Our patient developed gray hair from age 20 years, but microscopic examination lacked typical GS features (large uneven clumps of pigment) (data not shown). Furthermore, in contrast to typical GS patients that display hyperpigmented oval melanocytes without adjacent tissue pigmentation [18], a skin biopsy from the patient indicated normal distribution of melanin throughout the epidermis (Fig. 1E). RAB27A-deficiency is associated with defective cytotoxic lymphocyte exocytosis [10, 26]. Patient NK cells as well as CD8^+^CD57^+^ T cells displayed reduced exocytosis (Fig. 1F; normal range (mean ± 2SD) for induction of CD107a on NK cells was for K562 cell or anti-CD16 stimulation 9–41% and 30–66%, respectively, and for that on CD8^+^CD57^+^ T cells 28–76%, in healthy adults), but not abolished as frequently observed in FHL [3, 7]. Furthermore, in the patient, both NK cells and CD8^+^CD57^+^ T cells undergoing exocytosis displayed low intensity of CD107a surface expression, as previously reported in a patient with hypomorphic *UNC13D* variants associated with late-onset HLH [36].

Thus, the RAB27A p.R184Q was expressed at the protein level. Furthermore, evaluation of the patient suggested that the *RAB27A* variant may not affect melanosome trafficking of pigment but impair lymphocyte exocytosis.

### Rab27a p.R184Q Displays Unperturbed Function in Melanocytes While It Leads to Defective Cytotoxic Function in Lymphocytes

**Fig. 2:**
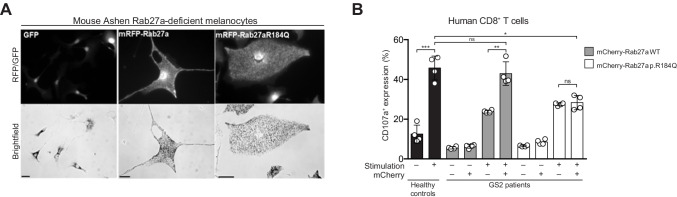
Reconstitution of RAB27A-deficient melanocytes and T cells with RAB27A WT and p.R184Q variants. **A** *Rab27a*-deficient mouse *ashen* melanocytes transduced with adenoviruses encoding mRFP-tagged RAB27A WT or p.R184Q variants. Fluorescence images show expression of vector control GFP or mRFP-RAB27A constructs and brightfield images melanosome distribution in transduced cells (bar indicates 20 μm for GFP and 10 μm for RFP images). **B** Unmanipulated CD8^+^ T cells from healthy control or RAB27A-deficient GS2 patients transduced with lentiviruses encoding mCherry-RAB27A WT or p.R184Q variants. Untransduced CD8^+^ T cells from healthy donors represent controls. For GS2 patient cells, the graph depicts the frequency of CD8.^+^ T cells with surface CD107a expression according to gating on mCherry expression, as indicated. Dots represent individual patients, bars represent mean values with SD. Statistics: ns non-significant *P* > 0.05; *P ≤ 0.05, **P ≤ 0.01

To understand if the *RAB27A* p.R184Q variant could cause disease, we evaluated its function in melanocytes and lymphocytes. Adenoviral *RAB27A* wild-type or p.R184Q variant constructs with an N-terminal mRFP fluorescent tag were generated for expression of RAB27A in melanocytes. These constructs were expressed in melanocytes from *ashen* mice that are homozygous for a *Rab27a* variant that disrupts exon splicing [41]. The RAB27A p.R184Q variant rescued pigment dispersion in Rab27a-deficient melanocytes in a manner comparable to RAB27A wild-type constructs (Fig. 2A). Furthermore, to evaluate if the patient-derived RAB27A variant could rescue lymphocyte exocytosis, we selected GS2 patients with biallelic *RAB27A* variants that resulted in defective RAB27A expression (Suppl Table 1) [32] and isolated peripheral blood CD8^+^ T cells and transduced them with lentiviral constructs encoding either N-terminal mCherry tagged RAB27A wild-type or p.R184Q proteins. After transduction, exocytosis was evaluated following anti-CD3 antibody stimulation. Untransduced CD8^+^ T cells from healthy volunteers demonstrated a robust increase in exocytosis upon anti-CD3 stimulation. The transduction efficiency of the mCherry-RAB27A WT constructs was higher in GS2 patient CD8^+^ T cells in all individuals (Suppl Fig S1A, S1B, S1C). The expression levels of the mCherry-RAB27A WT relative to mCherry-RAB27A p.R184Q were also consistently higher in GS2 patient CD8^+^ T cells in all individuals (Suppl Fig S1D). Importantly, anti-CD3 antibody stimulation significantly increased exocytosis by GS2 patient CD8^+^ T cells transduced with RAB27A wild-type, but not those with RAB27A p.R184Q constructs (Fig. 2B).

Taken together, these results support the notion that RAB27A p.R184Q facilitates melanosome pigmentation but does not efficiently support cytotoxic lymphocyte exocytosis.

### Altered Effector SLP2A/MUNC13-4 Binding Affinity for RAB27A p.R184Q

**Fig. 3:**
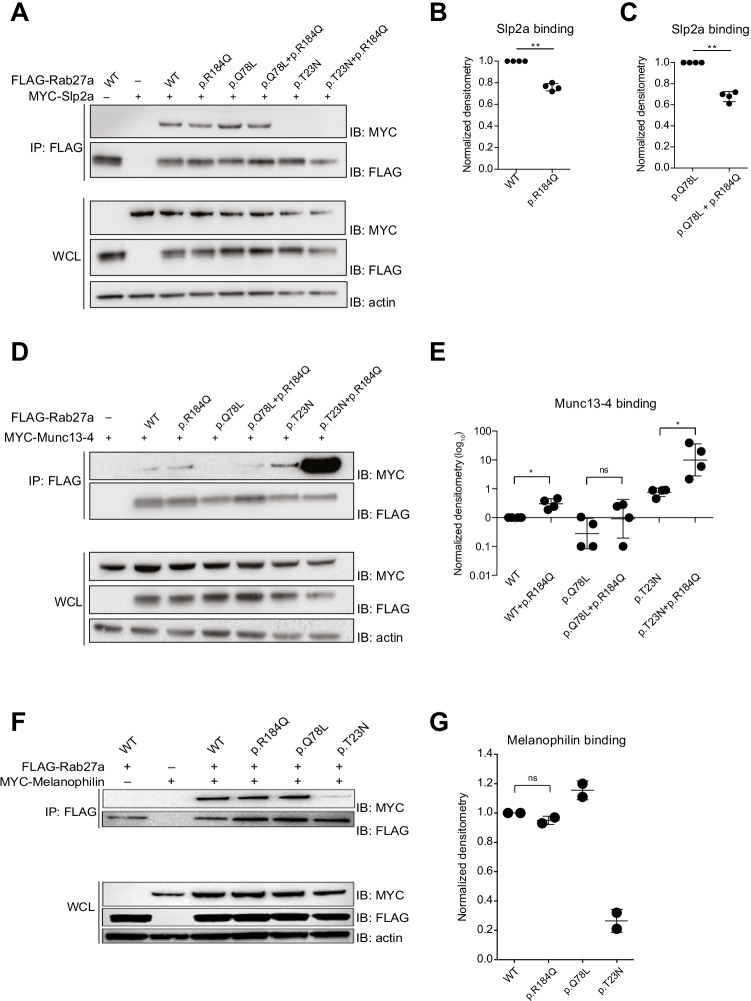
RAB27A p.R184Q displays altered binding to effector proteins present in immune cells. **A** 293FT cells co-transfected with MYC-SLP2A and FLAG-RAB27A WT p.Q78L (active mutant) or p.T23N (inactive mutant) in combination with patient-derived p.R184Q variant, as indicated. Immunoprecipitates (IPs) or whole cell lysates (WCLs) were probed by western blotting (IB) with antibodies, as indicated. **B** Quantification of SLP2A binding following anti-FLAG immunoprecipitation for RAB27A p.R184Q variant. **C** Quantification of Slp2a binding following anti-FLAG immunoprecipitation for Rab27a p.Q78L + p.184Q constructs. **D** 293FT cells co-transfected with MYC-MUNC13-4 and FLAG-RAB27A WT, p.Q78L (active mutant), or p.T23N (inactive mutant) in combination with patient-derived p.R184Q variant, as indicated. **E** Quantification of MUNC13-4 binding following anti-FLAG immunoprecipitation for the different RAB27A constructs in transfected HEK-293FT cells. **F** 293FT cells co-transfected with MYC-MELANOPHILIN and FLAG-RAB27A WT, p.Q78L (active mutant) or p.T23N (inactive mutant) in combination with patient-derived p.R184Q variant, as indicated. **G** Quantification of MELANOPHILIN binding following anti-FLAG immunoprecipitation for different RAB27A constructs in transfected HEK-293FT cells. Data are representative of at least three independent experiments, except in **G**, which displays results from two independent experiments. Statistics: ns, non-significant *P* > 0.05; *P ≤ 0.05, **P ≤ 0.01

To determine how the patient-derived RAB27A variant might interfere with lymphocyte exocytosis, we assessed the capacity of the RAB27A p.R184Q variant to interact with the effector proteins expressed in immune cells. FLAG-tagged RAB27A wild-type, “active” p.Q78L, or “inactive” p.T23N constructs, encoding the wild-type or p.R184Q variant, were co-expressed with plasmids encoding MYC-tagged, full-length SLP2A, MUNC13-4 in HEK-293FT cells. Co-immunoprecipitation of SLP2A, MUNC13-4 with tagged RAB27A variants was quantified in cell lysates (Fig. 3A). Relative to RAB27A wild type, the RAB27A p.R184Q variant displayed around 25% reduced binding to SLP2A (Fig. 3A, B). A reduction of more than 30% was observed when the RAB27A p.R184Q variant also carried the constitutive p.Q78L mutation (Fig. 3A, C). Furthermore, relative to RAB27A wild type, the RAB27A p.R184Q variant displayed tenfold increased binding to MUNC13-4 (Fig. 3D, E). The RAB27A p.R184Q variant also carrying the p.T23N mutation displayed 100-fold greater MUNC13-4 binding, whereas the p.Q78L mutation construct displayed only mildly increased MUNC13-4 binding (Fig. 3D, E). In contrast to previously published reports, the inactive RAB27A p.T23N mutant bound MUNC13-4 with higher propensity than the active p.Q78L mutant in our experimental setting (Fig. 3D, E). Similar co-immunoprecipitation experiments of MUNC13-4 in cells expressing melanophilin revealed equal binding of RAB27A WT and p.R184Q to melanophilin (Fig. 3F), whereas the constitutive active RAB27A p.Q78L variant displayed increased and the inactive p.T23N variant displayed decreased binding, respectively (Fig. 3F).

In summary, relative to RAB27A WT, the RAB27A p.R184Q variant displayed decreased binding to SLP2A and increased binding to MUNC13-4. This data suggests that the RAB27A p.R184Q variant displays an imbalance in effector binding, specifically disrupting MUNC13-4-mediated exocytosis.

## Discussion

Biallelic loss-of-function variants in *RAB27A* cause hypopigmentation and development of HLH [26], but atypical forms of GS2 lacking hypopigmentation have also been described. *RAB27A* missense mutations that selectively impair RAB27A binding to MUNC13-4 or non-coding rearrangements affecting a lymphocyte-specific promoter have previously been identified in GS2 patients, selectively displaying immunological features of the disease [4, 29, 31, 39]. We describe an adult-onset HLH patient from consanguineous parents harboring a novel homozygous *RAB27A* c.551G > A p.(R184Q) variant. Our results suggest a novel mode of selective disruption of RAB27A function in hematopoietic cells, leaving pigment dispersion intact.

**Fig. 4:**
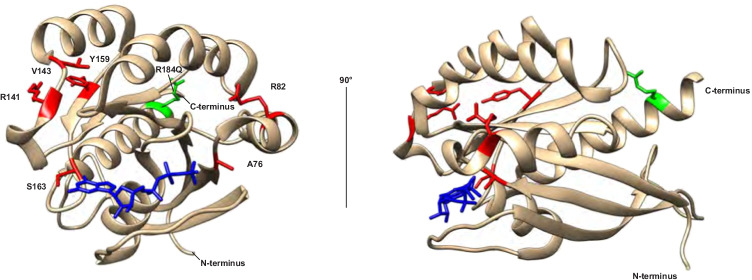
Contribution of the RAB27A α5-helix to effector protein interactions. Model of the RAB27A structure highlighting the R184 residue (green) located in α5-helix as well as other disease-causing RAB27A variants selectively associated with defective lymphocyte cytotoxicity but normal pigmentation that disrupt MUNC13-4 binding (red) (references #12, 13, 27). GTP is colored in blue

The structure of RAB27A p.Q78L variant in complex with the SLP2A has been solved (Fig. 4) [5], while RAB27A/MELANOPHILIN and RAB27A/MUNC13-4 complexes have not been reported. SLP2A interacts with the RAB27A α5-helix where the R184 residue is located [5]. The RAB27A R184 residue maintains electrostatic stability required for Slp2a binding, potentially explaining why exchange of charge impaired SLP2A binding in our experiments. The N-terminus of RAB27A can bind MELANOPHILIN, with the Rab27b/melanophilin structure indicating that the β1/β2-sheets and α2-helix of the closely structurally related RAB27A likely mediate binding of MELANOPHILIN [20]. A few HLH-associated *RAB27A* variants in GS2 patients with normal pigmentation selectively abolish MUNC13-4 but not MELANOPHILIN binding (Fig. 4) [4, 29, 31]. The RAB27A p.R141_V142delinsI and p.Y159C variants have indicated that the α4-helix may interact with MUNC13-4 [4]. Remarkably, our data indicates that the RAB27A p.R184Q variant binds MUNC13-4 significantly more strongly than RAB27A WT, with the affinity further increased by combination with the RAB27A p.T23N mutation predicted to mimic a GDP-bound inactive confirmation. MUNC13-4 was originally identified as an effector of GTP-bound RAB27A [38], and active RAB27A p.Q78L bound MUNC13-4 more strongly than inactive RAB27A p.T23N in the NK cell line YTS [23]. In our experiments in transfected HEK-293FT cells, RAB27A p.Q78L displayed higher binding to MELANOPHILIN and SLP2A than to RAB27A p.T23N, as expected. However, surprisingly, MUNC13-4 displayed higher binding to RAB27A p.T23N than to RAB27A p.Q78L. The combination of the RAB27A p.T23N and p.R184Q variants leads to a dramatic increase in binding, suggesting that an inactive, patient-derived RAB27A variant may be exceedingly efficient at binding and potentially sequestering MUNC13-4. Our results warrant further studies into the interplay between RAB27A binding to effectors SLP2A versus MUNC13-4 in the context of nucleotide binding, and how the affinities of these interactions may determine the efficiency of cytotoxic granule exocytosis and lymphocyte cytotoxicity.

Our results show an inability of patient-derived RAB27A p.R184Q to rescue exocytosis by RAB27A-deficient CD8^+^ T cells. RAB27A is required for docking and priming of the cytotoxic granules via interactions with SLP2A and MUNC13-4 [9, 12]. It is not clear what may contribute the most to the patient phenotype, (i) reduced expression of RAB27A p.R184Q relative to RAB27A WT in lymphocytes, (ii) decreased binding to SLP2A, (iii) increased binding to MUNC13-4, or a combination of these three factors. In 293FT cells, RAB27A WT and p.R184Q were similarly expressed, while RAB27A p.R184Q displayed lower expression in primary human CD8^+^ T cells. These data suggest a reduced stability of the patient-derived RAB27A variant in a physiological setting. Still, the reduced level of RAB27A is unlikely to fully explain the severe reduction in lymphocyte exocytosis. Overexpression of a SLP2A Slp homology domain construct has revealed an important role for RAB27A–SLP family protein interactions for CD8^+^ T cell granule exocytosis [12, 25]. In our biochemical experiments, the reduction of RAB27A binding to SLP2A was quite modest and may thus not explain the strong impairment in cytotoxic lymphocyte degranulation. Ménasché and colleagues demonstrated that overexpression of RAB27A p.Q78L in a CD8^+^ T cell line diminished granule exocytosis [24]. Thus, active RAB27A or strong RAB27A-MUNC13-4 interactions may result in decreased granule exocytosis and target cell killing. A priori, strong binding between RAB27A and MUNC13-4 leading to sequestration of MUNC13-4 might be expected to cause dominant forms of disease. The observations in this family, so far, do however not suggest a dominant mode of inheritance. Hopefully, identification of additional patients and families with this RAB27A variant can shed light on this important question. These results hopefully can spur further studies of the interaction of RAB27A with its distinct effectors.

Presenting at 35 years of age, to the best of our knowledge, this patient may represent the latest onset of GS2 reported to date [37, 39]. Directions on clinical penetrance of the *RAB27A* c.551G > A p.(R184Q) variant are lacking in this late-onset HLH patient. The family encompassed eight siblings, six of which did not consent or were not available to genetic testing. Further analyses of this family or other individuals homozygous for this variant that impairs RAB27A function in lymphocytes can hopefully provide further insights into the clinical penetrance. Nonetheless, the low cytotoxic T and NK cell exocytosis in the patient, the failure of the patient-derived RAB27A variant to reconstitute T cell exocytosis, and the degree of aberrant binding of RAB27A to MUNC13-4 suggest a significant impact of this variant on attenuating lymphocyte cytotoxicity and causing hyperinflammation. Similarly, autosomal loss-of-function *PRF1* missense mutations that severely impair perforin expression and lymphocyte cytotoxicity have been associated with development of HLH [6]. Notably, the patient also carried rare homozygous *VPS13B* missense and heterozygous *CARD11* missense variants of uncertain significance. The patient did however not display typical clinical features of Cohen syndrome associated with autosomal recessive VPS13B deficiency [17]. The clinical phenotype of heterozygous gain of function *CARD11* variants causes BENTA, a disease with susceptibility to viral infections and occasionally HLH [22], but *CARD11* variants previously associated with BENTA have been localized to the N-terminal domains of the protein whereas the *CARD11* variant in our patient was located at the C-terminus. Nonetheless, we cannot exclude that these variants in genes also expressed in immune cells might have modified disease in our patient.

In conclusion, our results indicate that the HLH patient–derived RAB27A p.R184Q variant maintains melanin distribution, yet displays dysregulated interactions with MUNC13-4 and SLP2A that impaired lymphocyte cytotoxicity. As such, this variant represents the first disease-associated RAB27A variant with increased MUNC13-4 binding. Together, these results suggest that the RAB27A p.R184Q variant can predispose to disease, potentially explain late-onset HLH in our patient, and advance insight into protein interactions causing pathophysiology. In addition, this case highlights the relevance of genetic testing in adults for relapsing HLH patients, especially when associated with a chronically active EBV infection or other immune anomalies. Further studies are warranted to develop rationale for targeted drug therapy.

## Supplementary Information

Below is the link to the electronic supplementary material.

Supplementary file1 (PDF 610 KB)

Supplementary file2 (PDF 95 KB)

## Data Availability

Data and materials generated during the current study are available from the corresponding author on reasonable request.

## Code Availability

Not applicable to this study.
