# Supplementary figures and images for: Novel RAB27A Variant Associated with Late-Onset Hemophagocytic Lymphohistiocytosis Alters Effector Protein Binding
Source: J Clin Immunol. 2022 Jul 23;42(8):1685–95. doi: 10.1007/s10875-022-01315-4 (PMC9700621; doi:10.1007/s10875-022-01315-4)

**A**

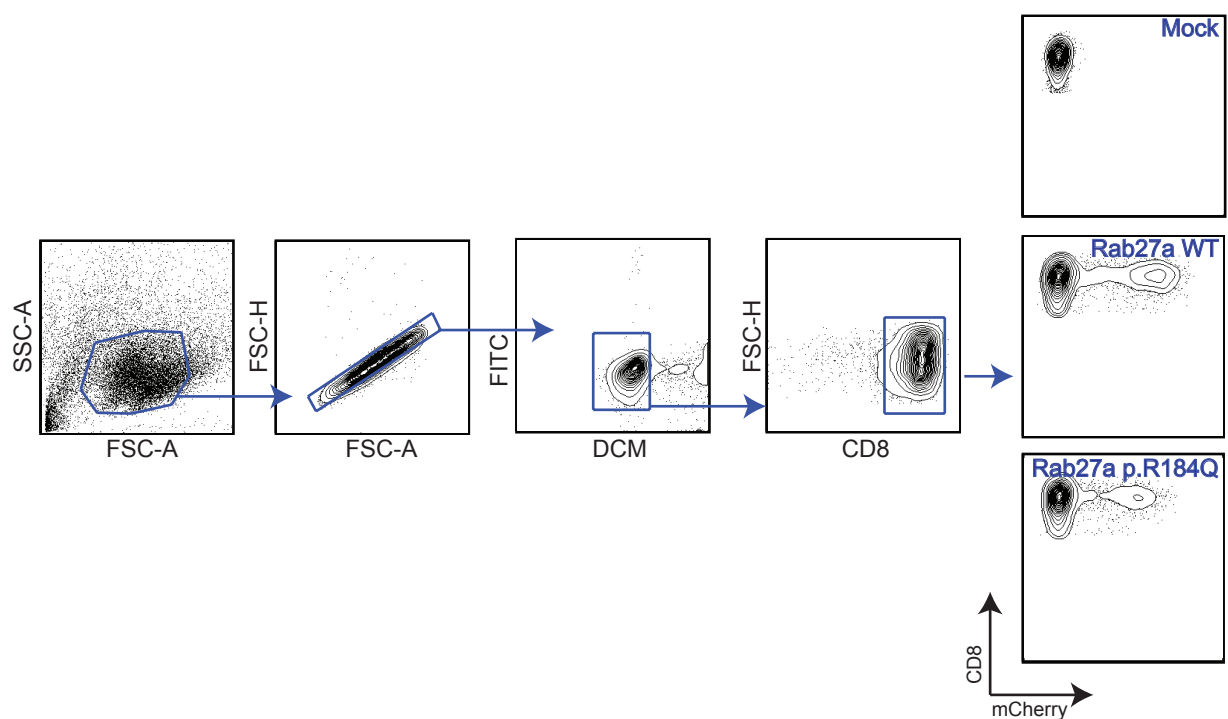

**B**

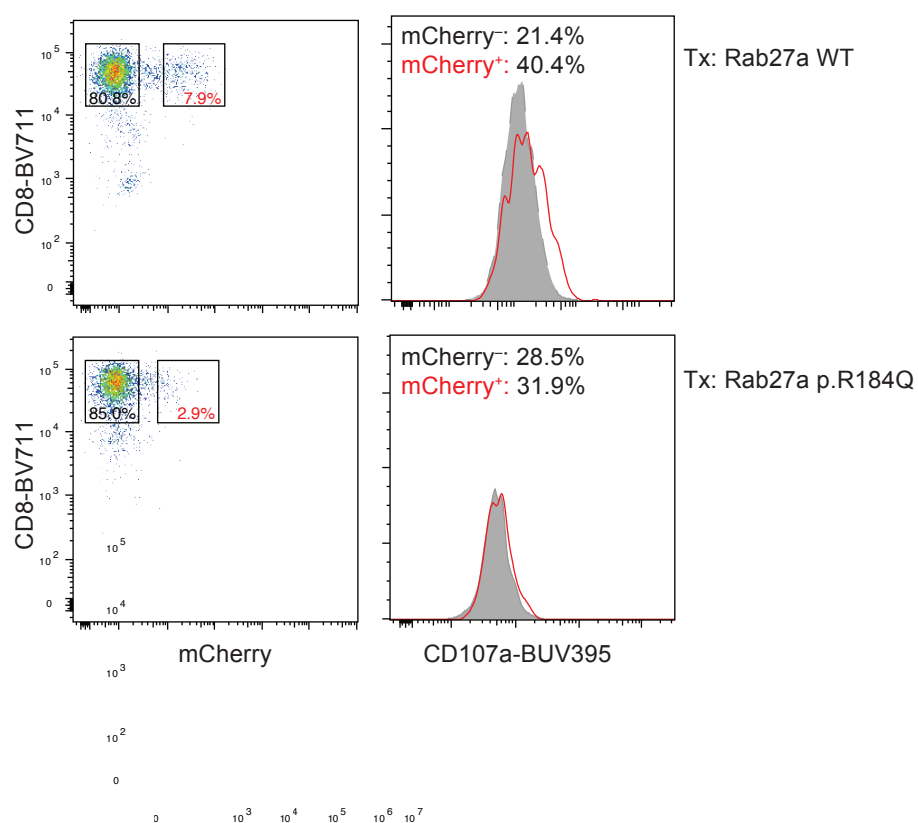

**C**

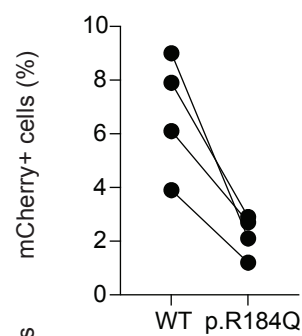

**D**

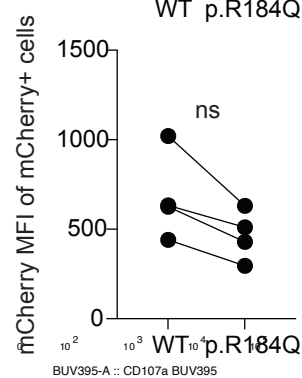

Supplement: Supplementary file 1 — Supplementary file1 (PDF 610 KB) [file 10875_2022_1315_MOESM1_ESM.pdf]
