# Supplementary material for: Novel RAB27A Variant Associated with Late-Onset Hemophagocytic Lymphohistiocytosis Alters Effector Protein Binding
Source: J Clin Immunol. 2022 Jul 23;42(8):1685–95. doi: 10.1007/s10875-022-01315-4 (PMC9700621; doi:10.1007/s10875-022-01315-4)
Supplement: Supplementary file 2 — Supplementary file2 (PDF 95 KB) [file 10875_2022_1315_MOESM2_ESM.pdf]

SUPPLEMENTAL TABLES AND FIGURE LEGENDS
Zondag, Torralba-Raga et al.

Supplemental Table 1. Genetics of the RAB27A-deficient patients used for lentiviral transduction

Patient	Gender	*RAB27A* variants	Diagnosis
**1**	M	Hmz c.514-518delCAAGC; p.Gln172NfsX2	GS2
**2**	F	Hmz c.514-518delCAAGC; p.Gln172NfsX2	GS2
**3**	M	Hmz c.148-149delAGinsC; p.Arg50GlnfsX35	GS2
**4**	F	Hmz c.514-518delCAAGC, p.Gln171NfsX2	GS2

FIGURE LEGENDS

Sup. Fig. 1. Flow cytometry gating strategy for transduced CD8$^+$ T cells. CD8$^+$ T cells from GS2 patients as well as healthy controls were isolated, transduced with lentiviruses encoding mCherry-tagged RAB27A constructs and then stimulated for evaluation of T cell receptor triggered degranulation. (**A**) Plots show the gating strategy for CD8$^+$ T cells from one representative patient. Plots to the right depict the transduction efficiency as revealed by mCherry expression for the RAB27A WT and p.R184Q constructs. (**B**) Plots show gating of CD8$^+$ T cells from GS2 patients based on mCherry expression (left column) and CD107a surface expression following anti-CD3 stimulation (right column) of cells transduced with RAB27A WT and p.R184Q constructs, respectively, from one representative GS2 patient. (**C**) Quantification of the frequency of mCherry$^+$ (high expression) CD8$^+$ T cells following lentiviral transduction of cells from four GS2 patients with different RAB27A constructs, as indicated. (**D**) Quantification of the MFI of mCherry$^+$ (high expression) CD8$^+$ T cells following lentiviral transduction of cells from four GS2 patients with different RAB27A constructs, as indicated.
